# Antisecretory factor as add-on treatment for newly diagnosed glioblastoma, IDH wildtype: study protocol for a randomized double-blind placebo-controlled trial

**DOI:** 10.1186/s13063-025-08792-z

**Published:** 2025-03-13

**Authors:** Erik Ehinger, Anna Darabi, Edward Visse, Charlotte Edvardsson, Gregor Tomasevic, David Cederberg, Sara Kinhult, Anna Rydelius, Christer Nilsson, Mattias Belting, Johan Bengzon, Peter Siesjö

**Affiliations:** 1 Department of Neurosurgery, Skåne University Hospital, Lund, Sweden; 2 Glioma Immunotherapy Group, Section of Neurosurgery, Department of Clinical Sciences, Lund University, Lund, Sweden; 3 Department of Clinical Sciences, Oncology, Lund University, Lund, Sweden; 4 Department of Immunology, Genetics and Pathology, Science for Life Laboratory, Uppsala University, Uppsala, Sweden; 5 Department of Hematology, Oncology and Radiophysics, Skåne University Hospital, Lund, Sweden; 6 Department of Neurology, Skåne University Hospital, Lund, Sweden; 7 Kamprad Laboratory, Department of Clinical Sciences, Lund University, Lund, Sweden

**Keywords:** Glioblastoma, Glioblastoma, IDH wildtype, Newly diagnosed, Salovum, Antisecretory factor, Randomized clinical trial, Protocol

## Abstract

**Background:**

Glioblastoma, IDH wildtype is the most common primary malignant brain tumor in adults. Despite best available treatment, prognosis remains poor. Current standard therapy consists of surgical tumor removal followed by radiotherapy and chemotherapy with the alkylating agent temozolomide. Antisecretory factor (AF), an endogenous protein, may potentiate the effect of temozolomide and alleviate cerebral edema. Salovum® is an egg-yolk powder enriched for AF and is classified as a medical food in the European Union. Salovum® has shown preliminary clinical effect on glioblastoma in a recent pilot study. Here, we aim to assess if add-on Salovum® to temozolomide therapy can improve outcomes in patients with newly diagnosed glioblastoma.

**Methods:**

This is a multi-center, double-blinded, randomized, placebo-controlled phase II-III clinical trial to investigate superiority of Salovum® over placebo as add-on treatment for glioblastoma during concomitant and adjuvant temozolomide therapy. Patients with newly diagnosed glioblastoma that are planned for temozolomide treatment are screened for eligibility and randomized to receive Salovum® (*n* = 150) or placebo (*n* = 150). An interim analysis will be performed after 80 included patients to guide whether to continue or terminate. Primary endpoint is 12-month overall survival. Secondary outcome is 24-month overall survival.

**Discussion:**

This study will likely produce high-grade evidence to support or reject Salovum® as add-on treatment for glioblastoma.

**Trial registration:**

ClinicalTrials.gov NCT05669820. Registered on January 3, 2023.

**Supplementary Information:**

The online version contains supplementary material available at 10.1186/s13063-025-08792-z.

## Background and rationale

Glioblastoma is the most common malignant primary tumor of the brain. Despite our best efforts, prognosis remains dismal with a median survival of 7–18 months [1]. Current standard therapy consists of surgical resection followed by radiotherapy and chemotherapy with the alkylating agent temozolomide (TMZ) [2, 3].

Several different mechanisms are thought to contribute to the therapy resistance of glioblastoma. The infiltrative growth pattern makes surgical removal of all tumor cells impossible. Although surgical methods have been refined over the years, the challenge ahead lies not in surgical advancements, but in developing novel oncological or multimodal therapies. An issue when exploring new ways to treat glioblastoma is the heterogeneity of the disease, not only between different individuals, but also between regions within the tumor itself [4]. This intratumoral heterogeneity, which has been attributed to both the presence of cancer stem cells and dynamic epigenetic mechanisms, might explain the poor prognosis and rapid recurrence of glioblastoma; the tumor consists of different cellular populations which each might respond differently to therapy. Thus, there is no single druggable target, and the multitude of oncogenic pathways contribute to treatment resistance. The tumor microenvironment (TME) constitutes a complex array of cellular populations and tumor-supporting mechanisms. Glioma cells manipulate and recruit non-transformed cells to produce an advantageous TME for tumor growth. Innate and adaptive immune cells are modified to support tumor growth and to suppress an antitumor immune response. Crosstalk between these cell populations through complex signaling pathways contribute to tumor growth, angiogenesis, invasion, immunosurveillance escape, and therapy resistance [5].

In addition to multidrug resistance, glioblastoma, as most solid tumors, displays an elevated intratumoral interstitial fluid pressure (IFP) [6, 7]. The increased IFP disturbs blood flow within the TME, thereby inducing hypoxia as a driving force of angiogenesis and cancer progression. Elevated IFP may also provide a physical barrier for drug uptake to the tumor and by this mechanism further contribute to therapy resistance [7].

Antisecretory factor (AF) is an endogenous and essential protein with proposed antisecretory and anti-inflammatory properties [8]. There are currently two approved methods of increasing antisecretory factor levels in humans and animals: SPC-flakes (specially processed cereals) and Salovum®. SPC-flakes consist of oats subjected to malting, a hydrothermal treatment process. Consuming SPC-flakes stimulates endogenous production of AF [9]. Salovum® is a freeze-dried egg yolk powder based on eggs from hens fed with SPC-flakes and contains large amounts of AF [10]. Salovum® and SPC-flakes are classified as medical food by the European Union and can be bought without a prescription in pharmacies in Sweden. Salovum® has been used in clinical trials and case series for a variety of conditions, including diarrheal diseases [11–13], inflammatory bowel disease [9, 14, 15], Ménière’s disease [16–20], and traumatic brain injury [21, 22]. No adverse effects have been reported, even at high doses [13, 15, 23].

AF has been shown to reduce the elevated IFP in experimental models of solid tumors [24]. Ilkhanizadeh et al. demonstrated that exogenous administration of AF reduced IFP levels, increased drug uptake in the tumor, and inhibited tumor growth in glioblastoma-xenografted mice [25]. They also showed that SPC-flakes combined with TMZ successfully prevented tumor growth and increased overall survival at 120 days from 30 to 100% compared to TMZ monotherapy [25]. Our own results show that intratumoral delivery of an active AF peptide, AF-16, boosts the effect of intratumoral chemotherapy and immune reactivity in an experimental glioma model [26].

Glucocorticoids (GCs) play a major role in the clinical management of glioblastoma. In clinical practice, GCs are prescribed liberally to relieve neurological symptoms and brain edema induced by the tumor. There is, however, increasing evidence that GCs may have a negative impact on survival in glioblastoma patients [27]. Mechanistically, it has been proposed that GCs induce a gene signature in the tumor that contribute to a worse prognosis [28]. Long-term therapy with GCs is also associated with many unwanted side effects such as diabetes, myopathy, insomnia, anxiety, and infections. Salovum® has recently been shown to reduce increased intracranial pressure in two pilot studies on patients with traumatic brain injury [21, 22]. We hypothesize that Salovum®, by reducing tumoral IFP, might decrease the peritumoral edema in glioblastoma and thus alleviate the need for GCs. In addition, and importantly, AF might facilitate TMZ delivery to the tumor by the same mechanism.

We recently published a pilot study where newly diagnosed glioblastoma patients were treated with Salovum® as add-on to concomitant therapy. The safety and feasibility endpoints were met. As glioblastoma is a diagnosis with poor prognosis, and Salovum® appears to have no serious side effects and experimental data supports its use, this trial is clinically motivated.

### Objectives

This trial intends to evaluate if Salovum® as add-on to standard therapy in glioblastoma can improve survival compared to placebo egg yolk powder.

## Methods

### Trial design

This is a national investigator sponsored, prospective, multicenter, double-blinded, randomized, placebo controlled, phase II-III clinical trial with two parallel groups to assess superiority of Salovum® as add-on to standard treatment in glioblastoma. Allocation ratio is 1:1. Recruitment began in January 2024. Main study center is Skåne University Hospital, Lund, Sweden. Secondary study center is Linköping University Hospital, Linköping, Sweden.

### Trial population

Up to 300 patients with newly diagnosed glioblastoma will be enrolled. Patients with newly diagnosed glioblastoma who are discussed in the hospitals’ multidisciplinary treatment conference will be screened for inclusion. Eligible patients are informed and asked to participate prior to starting TMZ therapy, and consent is obtained by one of the investigators or the research nurses. The informed consent form includes use of participant data and biological specimens in ancillary studies. After 80 patients are included in this phase II-III trial, a formal interim analysis will be performed. The interim analysis will guide the investigators to either abandon or proceed to phase III, where an additional 220 patients will be included. If the interim implies that we proceed to phase III, an amendment to the ethical permit will be filed, see Ethics section.

### Inclusion criteria

The inclusion criteria are patients with newly diagnosed glioblastoma, IDH wildtype according to the 2021 WHO criteria; age between 18 and 75 years; tumor has been surgically resected; the multidisciplinary conference recommends temozolomide therapy with or without radiotherapy; and the patient is able to give informed consent.

### Exclusion criteria

The exclusion criteria are inability to give informed consent; egg yolk allergy; only needle biopsy and thus no tumor resection; and multidisciplinary conference recommends radiotherapy as monotherapy.

### Standard therapy

All patients will receive the current standard of care. Fractionated radiotherapy is delivered in 30 fractions at 2 Gy per fraction, 5 days a week for 6 weeks. Concomitant chemotherapy consists of TMZ at a dose of 75 mg/m^2^, administered 7 days per week from the first day of RT until the last, for a total of 40 days. After a 4-week break, patients receive adjuvant TMZ at a daily dose of 150–200 mg/m^2^ for 5 days in six cycles of 28 days. Older patients with good performance status and MGMT methylated tumors are sometimes allocated a shortened radiotherapy (40.05 Gy/15 fractions) with concomitant and adjuvant TMZ. If radiotherapy is contraindicated, monotherapy with adjuvant TMZ is prescribed. These two groups will be included as well as we hypothesize that Salovum® may potentiate TMZ efficacy.

Patients in good clinical condition are offered treatment with tumor treating fields.

### Ethics

This study was reviewed and approved by the Swedish Ethical Review Authority, no. 2022–00028-02, in accordance with the ethical standards of the Declaration of Helsinki. The ethical permit allows us to include the first 100 patients. If the interim analysis shows a positive trend, an amendment of the ethical application will be filed for ethical review of the phase III extension.

### Randomization

Patients included are allocated to Salovum® or placebo egg yolk powder at a ratio of 1:1. Permuted block randomization with blocks of 4–6 will be used and was compiled with R (https://www.R-project.org). To achieve balance between the centers, each study center has its own assigned block list. No stratification is performed. Each patient is assigned a unique consecutive study number. The study number is linked to a box with the same number that contains sachets of Salovum® or placebo egg yolk powder. Each sachet is also marked with the patient’s study number. The placebo and Salovum® powder as well as the packaging look and taste identical. Both patients and investigators are blinded. If a patient needs unblinding, a separate box containing envelopes marked with the patient study number can be accessed. The contents will reveal to which arm the patient was allocated. The investigator who is including the subject to intervention or placebo is unaware of the allocation, which can only be revealed by opening an opaque envelope marked only with the sequential patient study number. The contents of the envelope reveal the allocation.

The allocation sequence was generated by a statistician who is not involved in inclusion, data collection, or clinical evaluations.

The boxes are packed by the trial administrator.

### Enrollment

Patients are enrolled through the hospitals’ neuro-oncologic multidisciplinary conference. Formal inclusion is performed by the responsible research nurse and the clinically active investigators.

### Interventions

#### Active substance

The active substance is Salovum®, an egg yolk powder enriched for AF. It is produced by Lantmännen Functional Foods AB, Stockholm, Sweden and supplied for the trial.

#### Placebo

The placebo substance is egg yolk powder, which contains low amounts of AF. It will be made from freeze dried yolk and be identical to Salovum® in color, taste, texture, and smell.

#### Dosage and timing of intervention

The Salovum® and placebo powder is pre-packed in sachets of 11 g. The dose will be 11 g three times daily. Therapy will be initiated differently dependent on the recommended treatment regime. The following scenarios are possible:

1. Concomitant (60 Gy/30 fractions or shortened with 40.05 Gy/15 fractions) and adjuvant therapy planned: Salovum® or placebo will be initiated 2 days prior to start of concomitant therapy and administered 3 times daily until 14 days after termination of the concomitant therapy. During adjuvant TMZ cycles, Salovum® will be administered from 2 days before, during, and 2 days after each cycle, a total of 9 days for each cycle.
2. Adjuvant TMZ cycles as monotherapy planned: Salovum® or placebo will be administered 2 days before, during, and 2 days after each cycle, a total of 9 days for each cycle.

If TMZ is discontinued due to tumor progression, Salovum is also discontinued.

#### Dispensing

Salovum® or placebo egg yolk powder is dissolved in at least 100 ml liquid (e.g., juice, lemonade, chocolate milk) of the patient’s choice and ingested orally. The mixture must not be heated as the proteins may denature.

#### Protocol adherence and monitoring

Protocol adherence will be monitored by frequent visits in the outpatient clinic during treatment (Table 1). Enrolled patients will have access to a research nurse that is involved in the research group. The investigators will have continuous communication with the study sites as well as frequent site visits. An external independent body (Clinical Studies Sweden, Forum South) will monitor the quality of the study. Participants who are in too poor clinical condition to participate in follow-up visits will be monitored for survival.

**Table 1:**
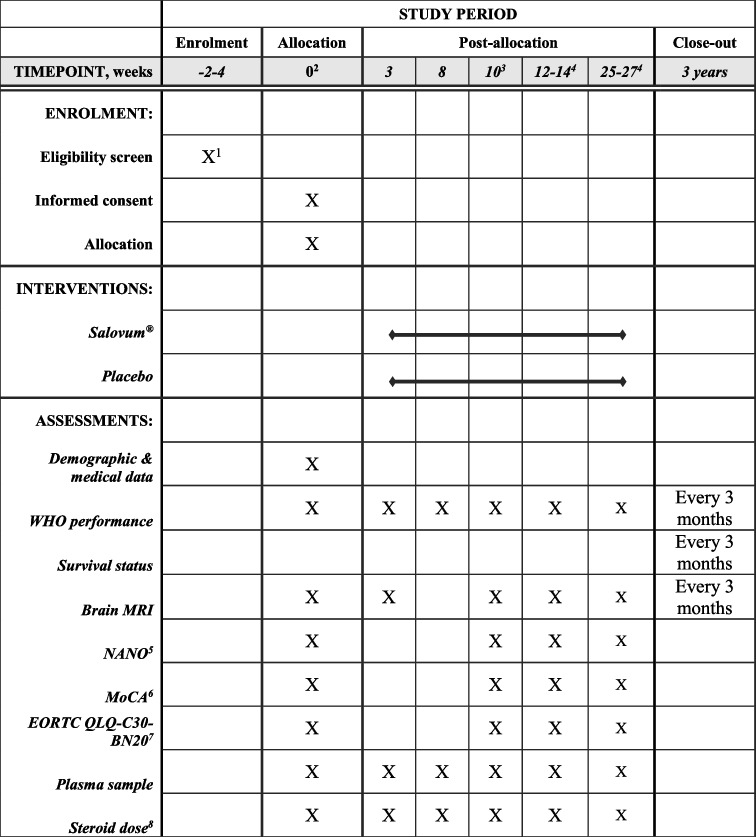
Schedule for follow-up visits. Day 0 is first day of Salovum® therapy

^1^Screening is performed at multidisciplinary treatment conference ^2^Concomitant treatment startup visit ^3^Post concomitant radiochemotherapy ^4^Post adjuvant temozolomide cycles 3 and 6 ^5^Neurological Assessment in Neuro-Oncology ^6^Montreal Cognitive Assessment ^7^European Organisation for Research and Treatment of Cancer Quality of Life Questionnaire C30 and BN20 ^8^Current daily steroid dose is registered, date and reason for dose changes are noted

### Study endpoints

The primary endpoint is the effect of Salovum® compared to placebo as add-on to standard therapy on 12-month survival in patients with glioblastoma, IDH wildtype. The secondary endpoint is 24-month survival. Exploratory endpoints include 6- and 12-month progression free survival, median OS and PFS, Kaplan–Meier and hazard ratio analysis of long-term survival. Survival time is defined as time from first surgery to death. Progression free survival is defined as time from first surgery to progression confirmed by consensus at a multidisciplinary conference (time to next intervention). Analyses will be made using chi-square and relative risk calculations. The patients will be followed for up to 5 years. Additional exploratory analyses and sub-group analyses may be performed by multivariable analysis with covariates such as age, performance status, extent of resection, molecular markers, center, steroid medication, tumor treating fields, and second-line treatment that will be published separately. Plasma will be drawn for analysis of inflammatory cytokines, blood lipids, brain damage markers, tumor derived exosomes, and additional relevant molecules.

### Data collection

Data will be collected in an electronic case report form (eCRF) only accessible by authorized personnel through two-step verification (REDCap). At baseline assessment, demographic and tumor data is collected. Cognitive status (Montreal Cognitive Assessment, MoCA, scale); neurological symptoms (Neurologic Assessment in Neuro-Oncology, NANO, scale); quality of life (EORTC QLQ-C30 and QLQ-BN20 questionnaires); steroid dependence; and radiological status (RANO) will be assessed. Patients will be monitored in the outpatient clinic according to Table 1.

### Biological specimens

Blood plasma is collected and stored in a valid Swedish biobank that is affiliated with the neurosurgical department. Specimens are primarily stored for future analysis in ancillary exploratory studies.

### Statistical power of endpoints

The power of the endpoints was calculated using data from our pilot study and a thoroughly reviewed Swedish glioblastoma database. An increase in 12-month overall survival from 67.3 to 87.5% (alpha = 0.05, beta = 0.2, power = 0.8) gives a sample size of 132 (66 + 66). An increase in 24-month overall survival from 19 to 40% gives a sample size of 146 (73 + 73). Increase in 6-month PFS from 71.4 to 87.5% gives a sample size of 196 (98 + 98).

### Handling of missing data

The patients are followed according to clinical routine, and data is documented in the patient charts as well as the eCRF. The amount of missing data is expected to be low. Patients who end participation before completing half of the scheduled trial intervention will be excluded and replaced by new patients. Patients who completed more than half of the prescribed trial intervention during concomitant radiochemotherapy will be counted as included. Included patients will be handled according to the intention-to-treat principle.

### Adverse events

Salovum® is a commercially available product and can be bought in pharmacies in Sweden without a prescription. As it has been available for many years and no toxicities have been reported, no serious adverse events are expected but adverse events will be monitored according to clinical routine and graded according to CTCAE version 5.0. If TMZ is discontinued because of toxicity, Salovum® or placebo will also be paused until TMZ can be reinstated.

### Interim analysis

A Data and Safety Monitoring Committee, consisting of at least one independent statistician and two independent clinicians with knowledge in the field, will perform an interim analysis after the 80th patient has been followed for 6 months. The DMC will assess the outcome based on 6-month PFS. If 6-month PFS differs more than 15% between the groups, the groups are unblinded. If placebo was superior, the study is terminated because of futility. If Salovum® is the superior group, the ethical permit will be amended to include 220 additional patients and continue to phase III. If 6-month PFS differs less than 15% between the groups, the groups are kept blinded and the ethical permit will be amended to include 220 additional patients and continue to phase III.

The 15% cutoff is based on data from the pilot study and our retrospective database. An increase in PFS from 71.4 to 87.5% (controls vs pilot group) is a difference of 18.4% or 16.1 percentage units.

### Communication of results

Results of the trial will be submitted for publication in a relevant journal after the clinical trial is finished, preferably in open access format.

## Discussion

Glioblastoma is an incurable disease, and despite major research efforts in the last decades all phase III trials have failed. The reason for the high failure rate is likely multifaceted. One major driving force may be that a large majority of phase II trials have traditionally not been RCTs, leading to a large amount of inadequate phase II trials that do not present sufficient information to decide whether to expand to phase III [29]. Also, long follow-up times to evaluate OS and PFS are inefficient. Conducting a phase III RCT is a huge undertaking and is ultimately a waste of time and resources if based on poorly designed phase I and II trials. A more effective rationale for selecting treatment modalities for phase III is warranted. We propose that rapidly evaluable phase II trials are a good alternative to solve this conundrum. Based on our power calculations from a Swedish database and our pilot study, we hypothesize that 6-month progression free survival data is sufficient to evaluate our intervention and guide whether to expand to phase III.

In glioblastoma research, patients included in clinical trials constitute a highly selected group of patients with a better prognosis than the general glioblastoma population [30]. Approximately half of glioblastoma patients are not eligible for most trials based on inclusion and exclusion criteria. We want our studied population to reflect clinical reality as much as possible. With this in mind, we open our trial for patients aged up to 75 and will include patients who because of comorbidities are recommended shorter concomitant regimes as well as TMZ monotherapy.

Salovum® is freeze-dried egg yolks and the doses we prescribe corresponds to eating approximately 4 egg yolks daily (220 kcal). The optimal dose is not known. In the pilot study, we prescribed higher doses (16 g × 4, approx. 420 kcal or 7 yolks daily). During the pilot study, we learned that some patients experienced an increased feeling of satiety and slight nausea when consuming such high amounts of Salovum®. We therefore decided to lower the dose.

As a result of radiochemotherapy, a transient increase in contrast enhancement is often seen on post-radiation magnetic resonance imaging. This benign contrast enhancement is often indistinguishable from tumor recurrence and is referred to as pseudoprogression. To accurately determine whether tumor has recurred, one must look at the whole clinical picture. To minimize the risk of erroneously declaring tumor progression, we define progression as radiological or clinical progression that has led to a change in the treatment regimen or initiation of second-line therapy, as decided by consensus at the hospitals’ multidisciplinary neuro-oncological conference.

## Trial status

The protocol is version 9, dated January 3, 2023. Recruitment began in January 2024. Recruitment is estimated to complete during 2026.

Supplementary information.

## Supplementary Information

Supplementary Material 1.

## Data Availability

The anonymized datasets will be available from the corresponding author on reasonable request.
